# Immersive virtual reality enables technical skill acquisition for scrub nurses in complex revision total knee arthroplasty

**DOI:** 10.1007/s00402-021-04050-4

**Published:** 2021-07-28

**Authors:** Thomas C. Edwards, Arjun Patel, Bartosz Szyszka, Alexander W. Coombs, Alexander D. Liddle, Rakesh Kucheria, Justin P. Cobb, Kartik Logishetty

**Affiliations:** 1 MSk Lab, Sir Michael Uren Biomedical Engineering Research Hub, Imperial College London, White City Campus, 86 Wood Lane, London, W12 0BZ UK; 2 Frimley Health NHS Foundation Trust, Frimley, UK

**Keywords:** Virtual reality, Simulation, Revision total knee arthroplasty, Patient safety

## Abstract

**Introduction:**

Immersive Virtual Reality (iVR) is a novel technology which can enhance surgical training in a virtual environment without supervision. However, it is untested for the training to select, assemble and deliver instrumentation in orthopaedic surgery—typically performed by scrub nurses. This study investigates the impact of an iVR curriculum on this facet of the technically demanding revision total knee arthroplasty.

**Materials and methods:**

Ten scrub nurses completed training in four iVR sessions over a 4-week period. Initially, nurses completed a baseline real-world assessment, performing their role with real equipment in a simulated operation assessment. Each subsequent iVR session involved a guided mode, where the software taught participants the procedural choreography and assembly of instrumentation in a simulated operating room. In the latter three sessions, nurses also undertook an assessment in iVR. Outcome measures were related to procedural sequence, duration of surgery and efficiency of movement. Transfer of skills from iVR to the real world was assessed in a post-training simulated operation assessment. A pre- and post-training questionnaire assessed the participants knowledge, confidence and anxiety.

**Results:**

Operative time reduced by an average of 47% across the 3 unguided sessions (mean 55.5 ± 17.6 min to 29.3 ± 12.1 min, *p* > 0.001). Assistive prompts reduced by 75% (34.1 ± 16.8 to 8.6 ± 8.8, *p* < 0.001), dominant hand motion by 28% (881.3 ± 178.5 m to 643.3 ± 119.8 m, *p* < 0.001) and head motion by 36% (459.9 ± 99.7 m to 292.6 ± 85.3 m, *p* < 0.001). Real-world skill improved from 11% prior to iVR training to 84% correct post-training. Participants reported increased confidence and reduced anxiety in scrubbing for rTKA procedures (*p* < 0.001).

**Conclusions:**

For scrub nurses, unfamiliarity with complex surgical procedures or equipment is common. Immersive VR training improved their understanding, technical skills and efficiency. These iVR-learnt skills transferred into the real world.

## Introduction

Adverse events in hospitals happen in approximately 10% of admissions [1–3]. They most commonly occur in the operating room (OR) and approximately half of these events are considered preventable [1, 4–6]. Error reduction strategies have focussed on improving the technical and non-technical performance of the OR team [7–9]. Superior non-technical or teamwork skills in the OR are associated with a reduction in adverse events [10], and improved patient outcomes including reductions in mortality and complications [11]. Despite evidence highlighting the importance of the whole surgical team in improving patient safety, structured technical training for scrub nurses is significantly lacking across the globe [12, 13].

The essential roles of a scrub nurse (also termed scrub technician or perioperative nurse) include the preparation and assembly of instrumentation, coordination between surgeon and unscrubbed team members, maintaining asepsis, and timely, efficient delivery of instrumentation to the surgeon. Despite being a pivotal part of the surgical team, scrub nurses have no speciality-specific structured training pathway in the United Kingdom, USA or Australia [12, 13]. The training they receive is often ad hoc, delivered by industry representatives prior to surgery or through ‘double-scrubbing’ alongside a more experienced scrub nurse. The result of learning in this unstructured manner, is high levels of anxiety, low confidence and concerns that patient safety is being adversely affected by their lack of knowledge or technical ability [12, 13]. Staff shortages, frequent rotation through specialities and time pressure when in theatre, further limit their ability to acquire the necessary sub-speciality skills to perform effectively in the OR. With a well-established correlation between prolonged operative time and an increased risk of developing significant complications [14–16], it is vitally important OR teams are as efficient as possible. Infrequently performed operations with multiple pieces of equipment or steps, therefore present a huge challenge. Targeted training for these operations could improve procedural knowledge and operative efficiency, thus protecting patient safety.

Although most surgical procedures require a well-choreographed interaction between the patient, surgeon and scrub nurse, some specialties, such as trauma and orthopaedics, require a more intricate knowledge of the complex equipment utilised. Complex procedures such as revision total knee arthroplasty (rTKA) have inventories exceeding 500 separate pieces of equipment [17]. Compared to primary TKA, revisions are more technically difficult, with greater intraoperative variability. They are performed less frequently, so intraoperative training opportunities are limited, and these operations have almost double the complication rate [18]. rTKA is therefore an ideal model to test novel training solutions for complex surgical procedures to improve scrub nurse performance and overall procedural efficiency.

Immersive virtual reality (iVR) simulation presents a potential cost-effective, innovative solution to better prepare scrub nurses for these difficult operations [13, 19, 20]. iVR combines a motion-tracked headset with hand-held controllers to place the user in an interactive virtual OR with a patient, staff members and surgical instrumentation. iVR has been validated for training junior surgeons in both open and endoscopic procedures—improving their technical knowledge, visuospatial skills, efficiency of movement, non-technical skills, while reducing operative time and errors [20–22]. However, it is largely untested for training scrub nurses for these critical skills [19, 21, 22].

This study aimed to investigate whether an immersive virtual reality curriculum can train scrub nurses to perform revision TKA. We hypothesised that the iVR curriculum would (1) improve procedural knowledge and reduce errors, (2) increase confidence and reduce anxiety levels, and (3) that these virtually learned procedural skills would transfer to the physical world.

## Methods

### Participants and setting

After obtaining institutional approval (Frimley NHS Trust Quality and Audit Department Ref: FH221), we recruited fifteen fully qualified scrub practitioners with at least 6 months’ experience in orthopaedics. They provided informed consent and were trained in iVR (Attune^®^ Revision TKA Simulator v1.1, Pixelmolkerei, Chur, Switzerland) to perform a revision TKA using a complex system where the full inventory contains 506 pieces of instrumentation (Attune^®^ Revision Total Knee System, DePuy, Warsaw, Indiana, United States) [17]. Training was delivered in a simulation centre at a university-affiliated hospital over four weeks incorporating four separate iVR sessions. Participants were excluded if they had scrubbed for more than five rTKA procedures using this equipment or if they had previously participated in immersive virtual reality surgical simulation training. They were not renumerated for participation in this study.

### Pre-simulation

Participants provided demographic information including hand dominance, previous clinical experience, virtual reality or video game experience and documented any previous training for rTKA. They were asked to rate their current levels of confidence and anxiety scrubbing for the operation as well as rate their knowledge of the procedural steps/equipment on a 5-point Likert scale (1 being no confidence, 5 being very confident). To ensure a baseline level of knowledge, participants were given a short lecture highlighting the basic principles of rTKA and strategies for how the instrumentation and implants could be used to address specific bone defects.

All participants then undertook a simulated real-world assessment to establish their baseline level of competence in rTKA. The assessment integrated knowledge and technical skills typical of those required during real surgery. It was designed through consensus between three expert surgeons, one expert scrub nurse, and two industry representatives from DePuy who are involved in the training of surgeons and scrub nurses. The real equipment was arranged on tables replicating the real operation. Reflecting the complex procedural sequence of a real rTKA, participants were asked to correctly select, assemble and supply equipment for the surgeon’s use, at 15 time points during the simulated surgery. A time limit of one minute per question was imposed to reflect the time pressured real operating theatre. Assessments were filmed and marked by two assessors independently, if there were conflicts between allocated scores, these were resolved by discussion with a third assessor.

### iVR curriculum

**Fig. 1:**
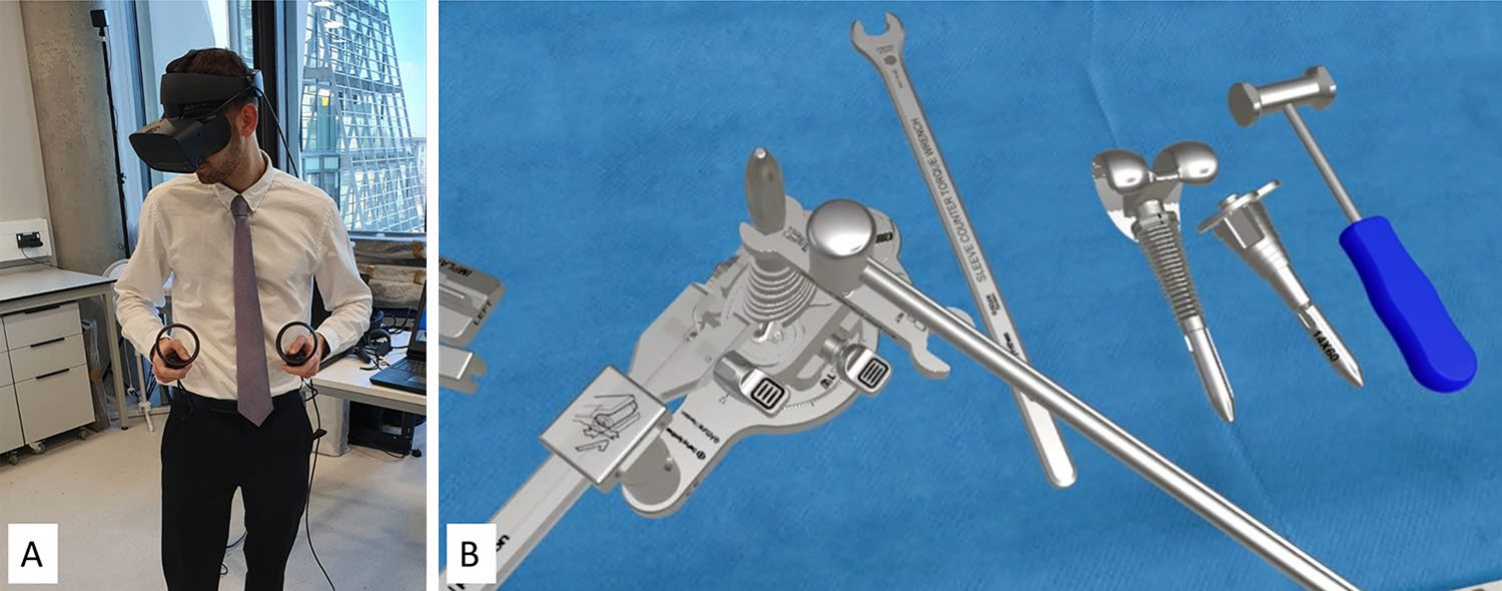
**A** Participant performing the iVR training with motion-tracked headset and controllers **B** participants view of the simulated rTKA equipment through the iVR headset

All participants completed a four-session immersive virtual reality curriculum with a minimum of 2 and a maximum of 10 days between sessions. This involved wearing a VR headset and performing procedures using motion-tracked controllers (Fig. 1). Initially, participants completed a non-surgical iVR orientation exercise to familiarise themselves with the controllers and iVR environment. The iVR software uses on-screen prompts and commentary to guide participants through performing a rTKA for a patient with an existing primary TKA. The user performs the roles of both the scrub nurse (selection, assembly and supply of the instrumentation, and subsequent disassembly) and the surgeon (surgical approach, application of instrumentation to the patient, and performing of bony cuts to the femur and tibia). There were bone defects in both the distal femur and proximal tibia requiring the use of specialist metal sleeves, stems and modular components. The operation started after the primary implants were removed. All sessions were supervised by a simulation technician, who ensured safety and provided technical support. The sessions varied in duration as participants progressed; however, no session lasted longer than 2 h.

### iVR training and assessment

In the first training session, participants performed the operation with the iVR automated guide and prompts. In the second, third and fourth sessions, participants performed the guided operation, before then performing it again in an ‘assessment mode’. The assessment mode provided no guide or commentary, and participants were automatically prompted for the next step if progress had not been made after 30 s. The iVR performance of participants was recorded as a computer-generated video of their avatar performing virtual surgery.

### iVR outcome measures

Participants’ performance in iVR was analysed by two independent assessors using event tagging software (Behavioural Observation Research Interactive Software [23]). They measured (1) procedural duration, recorded from skin incision to final implantation, (2) the number of times the user required an assistive prompt, and (3) the number of errors made in instrument selection or use. The distance moved by the user’s hands and head were also recorded automatically by the computer software, as efficiency of movement is correlated with surgical proficiency [24].

### Post-simulation

Following the final iVR session, participants repeated the real-world simulated assessment using the real equipment. An additional five questions were incorporated to reduce recall bias. Participants’ performance was compared to their pre-simulation assessment. Participants then repeated the questionnaire reflecting on their confidence, anxiety and knowledge since iVR training.

### Statistical analysis

Statistics were performed in Stata/IC 10.1 (StataCorp LP, College Station, TX). Data were first tested for normality using the Kolmogorov–Smirnov test and visually inspecting the data using histograms and *q*–*q* plots. Where normally distributed, and the assumption of sphericity (as determined by Mauchly’s test) was not violated, a one-way repeated-measures analysis of variance (ANOVA) test was used to compare performance across the three in VR assessments. Where non-parametric, Friedman test was used; and where sphericity was violated, the Greenhouse–Geisser correction was applied. In circumstances where missing data values were present, a mixed-effects model analysis was employed. Paired *t*-tests were used to compare differences in pre- and post-simulation physical world assessments and questionnaire results. *p*-values < 0.05 were considered significant. Strength of agreement between the video avatar assessors was determined using a two-way, mixed-effects model intraclass correlation co-efficient (ICC) for consistency of agreement. Typically, values > 0.75 indicate good agreement [25]. Mean values between the two assessors were used in the analysis.

## Results

### Demographics

Fifteen scrub nurses were initially recruited, 5 (30%) were unable to complete the full 4-week curriculum (3 nurses declined further participation after experiencing temporary dizziness during their first iVR training, and 2 were unavailable to attend the final session due to staff shortages). The key demographics are shown in Table 1. The mean age of the 10 remaining participants was 33.3 ± 10 (range 27–58) years, all were right hand dominant with a mean of 6.4 ± 7 (range 0.5–25) years post graduate experience. Previous rTKA experience was similar, two participants had previously scrubbed for rTKA operations using this specific equipment for fewer than five surgeries.

**Table 1:** Demographics and experience

Variable	*n* = 10 (SD, range)
Age (years)	33.3 (10.0, 27–58)
Sex	
Male	7 (70%)
Female	3 (30%)
Clinical experience (years)	6.4 (7.0, 0.5–25)
Hand dominance	
Right	10
Left	0
Video game experience	
Yes	3 (30%)
No	7 (70%)
Previous VR experience	
Yes	2 (20%)
No	8 (80%)

*VR* virtual reality, *SD* standard deviation

### Questionnaire results

**Fig. 2:**
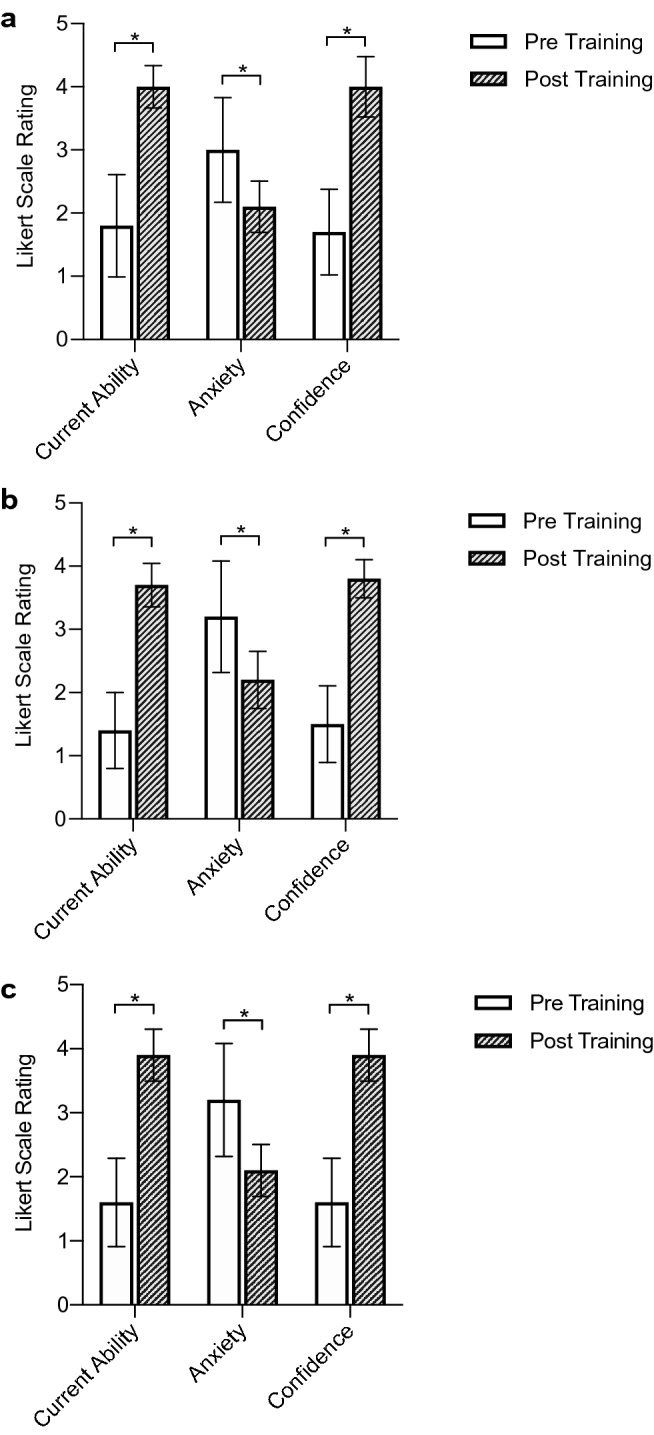
Bar graphs demonstrating qualitative feedback in participants self-reported ability, anxiety and confidence, both before and after iVR training relating to **A** identification and understanding what components are used for **B** assembly of components **C** sequence of steps during surgery. Error bars denote a 95% confidence interval, significant differences (*p* < 0.05) highlighted with an asterisk (*)

Following completion of the iVR curriculum, confidence as recorded on the 5-point Likert scale increased significantly in all areas (Fig. 2). Confidence increased in component assembly (mean pre-simulation 1.5 ± 0.85 to 3.8 ± 0.44 post-simulation, *p* < 0.001) and knowledge of the procedural steps (mean pre-simulation 1.60 ± 0.97 to 3.89 ± 0.60, *p* < 0.001). Levels of anxiety also reduced significantly in these areas (*p* < 0.001) as demonstrated in Fig. 2.

### iVR assessment

**Fig. 3:**
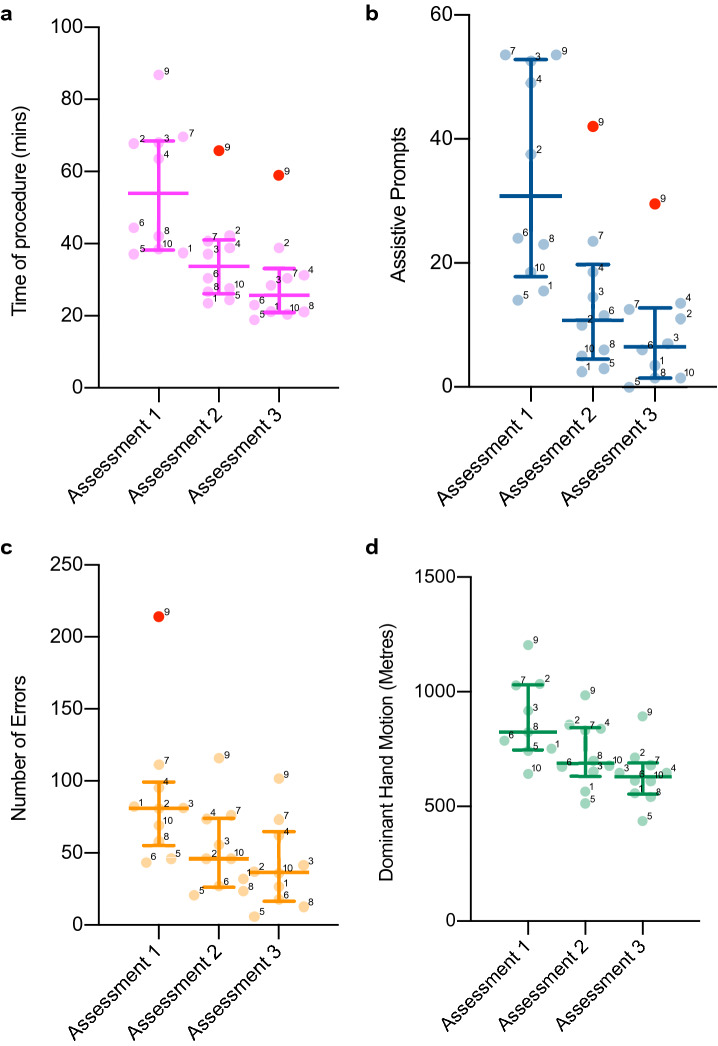
Column scatter graphs for iVR outcomes across the three unguided assessments—**A** operative time in minutes **B** assistive prompts **C** total errors committed **D** dominant hand motion in metres. The median is represented with the interquartile range. Individual data points labelled with participant identification number to the top right of the dot. Tukey outliers (data points 1.5 interquartile ranges above the 75th centile or below the 25th centile) demonstrated in red. For all outcomes, significant differences were detected between all of the assessment sessions (*p* < 0.05)

All participants improved across the three unguided assessment sessions (Fig. 3), reducing their operative time by 47% (mean 55.5 ± 17.6 min in unguided Session 1 to 29.3 ± 12.1 min in Session 3, *p* > 0.001), assistive prompts by 75% (34.1 ± 16.8 to 8.6 ± 8.8, *p* < 0.001), dominant hand motion by 28% (881.3 ± 178.5 m to 643.3 ± 119.8 m, *p* < 0.001) and head motion by 36% (459.9 ± 99.7 m to 292.6 ± 85.3 m, *p* < 0.001). The total numbers of procedural errors committed in iVR reduced by 47% from a mean of 88 ± 49 in Session 1, to 41.4 ± 29.7, *p* < 0.001 in Session 3. Inter-observer reliability for video analysis was good for all measurement variables (ICC > 0.8) as demonstrated in Table 2.

**Table 2:** Inter-observer reliability of video-based analysis

Variable	Inter-observer reliability^a^
Prompts	1.00 (0.99–1.00)
Instrument selection error	0.98 (0.96–0.99)
Instrument use error	0.87 (0.74–0.93)
Total error	0.98 (0.96–0.99)

^a^Intraclass correlation co-efficient with 95% confidence interval in parentheses

### iVR learning curve

Significant differences were found between all three consecutive iVR assessments for operative time, prompts, errors and motion metrics (*p* < 0.05), indicating that on average no plateau was reached in the participants’ learning curves on any metric. The best performing participants, however, did start to plateau by the third assessment in procedural time, prompts and errors as demonstrated in Fig. 3.

### Real-world assessment

**Fig. 4:**
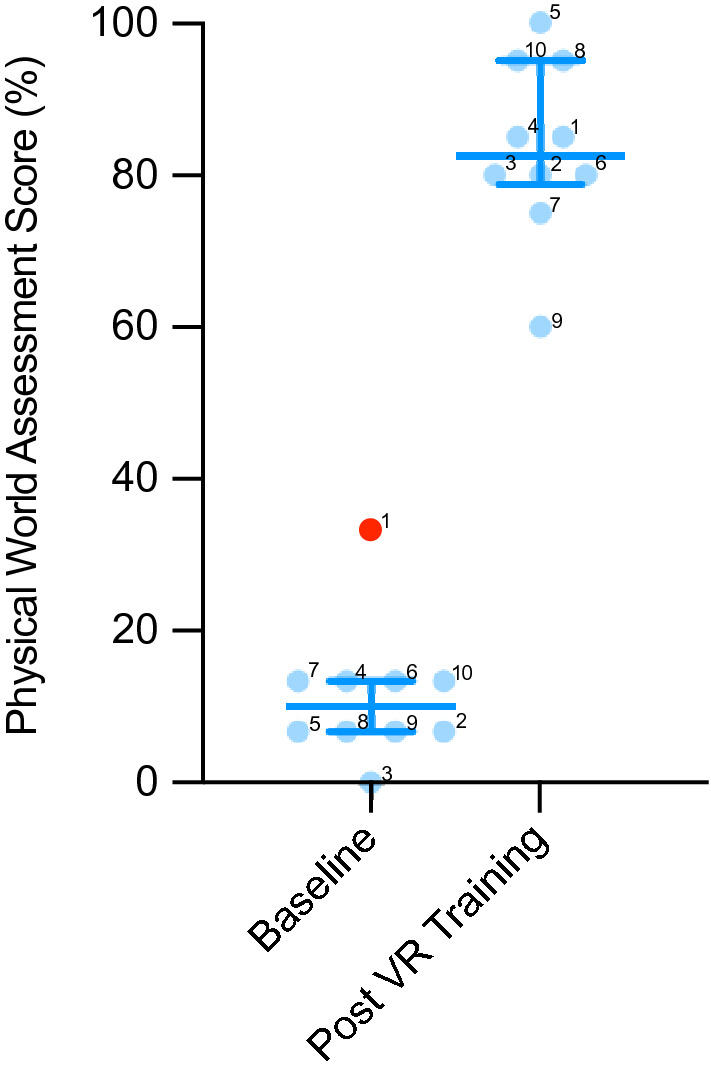
Column scatter graph for the real-world assessments before the iVR training (left) and after (right). The median is represented with the interquartile range. Individual data points labelled with participant identification number to the top right of the dot. Tukey outliers (data points 1.5 interquartile ranges above the 75th centile or below the 25th centile) demonstrated in red. The difference between the two assessments was significant (*p* < 0.001)

Real-world performance of rTKA improved from 11.3 ± 8.92% correct answers pre-training to 83.5 ± 0.12% after iVR training, *p* < 0.001 (Fig. 4). The highest performing and lowest performing participants in the iVR assessments were also the highest and lowest scoring in the physical world assessment (participants 5 and 9, Fig. 4).

## Discussion

An immersive Virtual Reality curriculum significantly improved scrub nurses’ skills, knowledge and efficiency in scrubbing for a revision total knee arthroplasty. Crucially, skills learnt in iVR were transferred to the real world, suggesting that this is a valid technique for improving the quality and safety of rTKA.

This study is unique in its measuring of the impact of immersive virtual reality on technical skills acquisition of complete operations for scrub nurses. Bracq et al. in a recent study demonstrated acceptability using a virtual reality mobile-app module to teach scrub nurses to set up the instrumentation table for craniotomy procedures [26]. The authors found scrub nurses interest to increase after using the simulator, finding it a useful and realistic way to learn. Similar to the present study, the authors commented that some participants developed simulator sickness and visual discomfort. In our study, two of the participants were unable to complete the full curriculum due to vestibular symptoms. This may present a reason for potential lack of early adoption of iVR training; however, advances in technology may reduce these side effects for future studies.

Although immersive virtual reality has not been fully utilised in training scrub nurses, a number of studies have demonstrated similar improvements in technical ability whilst training surgeons. In a study by Logishetty et al., 32 surgical residents were trained to perform total hip arthroplasty operations using an anterior approach [19]. Residents improved significantly over the 6 session curriculum reducing their in VR assistive prompts, errors and procedural times, reaching expert levels by the 4th session. Similar to the present study, these skills translated into the physical environment and the best performing residents on the VR assessments were also the highest scoring in the physical world assessment. This emphasises the construct validity of VR training in assessing procedural skills acquisition. A number of other studies have demonstrated a similar effect in training surgeons, further supporting this concept [20, 21].

With a substantial proportion of preventable adverse events occurring in operating theatres [1, 4], it is no surprise that a number of studies have focussed on assessing and improving teamwork in this environment. Evidence that better team performance reduces error and improves outcomes is growing. In a recent study by Fecso et al. focussing on technical performance in bariatric surgery, the authors report strong correlations between non-technical performance of both the surgical and nursing teams and the number of technical adverse events committed [10]. Mazzocco and colleagues further emphasised the importance of team performance observing team qualities in 300 operations [11]. The authors conclude that when good teamwork behaviours were observed infrequently, patients were more likely to experience death or a major complication. Furthermore, operating team efficiency appears to an important factor in optimising patient outcomes in arthroplasty surgery; a number of studies have linked prolonged operation times to an increased risk of developing a significant complications [14–16]. In a recent registry-based study including 92,343 TKA operations, operation times > 100 min were associated with almost double the risk of experiencing deep infection [16]. Similar findings have been demonstrated by other authors highlighting significant reductions in complications with shorter, more efficient operating times [14, 15]. In the present study the VR training almost halved the time taken for participants to complete the procedure. This substantial improvement in efficiency may therefore have a positive impact on patient safety, providing this observed effect translates into the real operating theatre. This presents an interesting avenue for future research.

To achieve optimal patient outcomes, the operating team must demonstrate excellent non-technical skills, and also have reached a high level of proficiency in their individual roles to avoid unnecessary delays or mistakes. Training of surgeons and anaesthetists is highly structured, with a competency-based curriculum and formal assessment before independent practice [27]. Scrub nurses, on the other hand, have very little formal training in the UK [12], with a similar situation noted in other countries [13]. With no formal undergraduate or postgraduate training, newly qualified scrub nurses are expected to pick up these specialised skills, observing and double scrubbing in the highly pressurised environment of the operating theatre [12]. As reported in an Australian study by Pupkiewicz et al., staffing shortages, frequent rotation to unfamiliar specialties and concerns over not being familiar with the many pieces of equipment required, provide further challenges to learning [13]. In the same study, novice scrub nurses reported high levels of anxiety, fear and lack of confidence in their abilities—many of the studied cohort being concerned their lack of knowledge of a particular operations may compromise patient care [13]. The authors mention the need to develop a ‘safe environment to learn and practice skills’ [13]. These findings have been supported by Radford et al. emphasising the training gap and the impact of service pressures on the learning process [12]. Immersive virtual reality meets this requirement, representing a highly cost-effective, easily implemented method for scrub nurses to engage in safe, repetitive practice in a realistic operating theatre environment [19]. Our study supports the notion that this may improve their confidence and reduces anxiety whilst imparting the necessary technical skills to perform efficiently in the physical world. Furthermore, the recent COVID-19 pandemic has placed limitations on staff numbers in the OR alongside travel restrictions, which has led to difficulties accessing help from industry representatives. This may exacerbate feelings of anxiety and increase stress, particularly for newly qualified scrub nurses or those unfamiliar with specialist equipment. Although changes in national teaching policy for scrub nurses are warranted, wide-spread adoption of iVR technology may go some way to address the deficit in scrub nurse training, having a likely knock-on effect on patient outcomes.

This study has several limitations. First, it was a single-arm study with small numbers and a relatively large loss to follow up. The cohort examined was specific being orthopaedic scrub nurses and we only focussed on one operation which may limit the generalisability of our results to other cohorts or operations. Whilst there was no control group, the participants acted as their own controls, having had a baseline assessment prior to the training. Second, given that the study took place over 4 weeks, we cannot guarantee that the training effect observed was entirely attributed to the iVR training. Although no participants scrubbed for a revision knee procedure during the study period, they may have sought out other training materials to aid their performance, such as the surgical technique manual. This may have biased our results. Third, we were not able to assess performance in the real-world operating theatre or link their performance to any patient outcomes. However, the physical world assessment used was designed to reflect the tasks required during a real operation and the real equipment was used. Another limitation was the analysis of the iVR videos for errors and prompts was not determined automatically by the computer software. Despite excellent inter-observer reliability for these measurements, we did not examine repeatability. If performed, this may have strengthened the validity of this data. Finally, some of the participants were still improving in the final training session and had not plateaued on the learning curve. Participants received four sessions based on previous iVR studies in hip arthroplasty showing plateau of skills in surgeons [19]. Studies of novices learning simulated arthroscopy have suggested five to six sessions are required before plateau of technical skills [28]. Based on the present study, it is unknown whether more sessions would have benefited scrub nurse participants learning rTKA. This combined with the lack of expert benchmarking, means we cannot comment on how many training sessions are sufficient to reach expert levels of proficiency.

## Conclusion

Immersive VR training improves scrub nurses understanding, technical skills and efficiency in complex revision knee arthroplasty surgery. These iVR-learnt skills appear to translate into the physical environment. The wide-spread adoption of this technology could address the need for a safe learning environment for scrub nurses to acquire these complex skills.
